# Nanomaterials and Nanodevices

**DOI:** 10.1155/2015/250401

**Published:** 2015-03-26

**Authors:** Xiao-Feng Zhao, Mu-Chun Wang, Jen-Ching Huang, You Qiang, In-Seok Yoon

**Affiliations:** ^1^Heilongjiang University, Harbin 150080, China; ^2^Minghsin University of Science and Technology, Hsinchu 300, Taiwan; ^3^Tungnan University, New Taipei City 222, Taiwan; ^4^University of Idaho, Moscow, ID 83844, USA; ^5^Induk University, Seoul 139-749, Republic of Korea

Nanoscience has been an emerging and rapidly expanding discipline in the past decade, which is the engineering of functional systems at the molecular scale. This covers both current work and concepts that are more advanced. Nanoscience and nanotechnology include three fields: nanomaterials, nanodevices, and nanomeasurement and nanocharacterization. This special issue mainly focuses on nanomaterials and nanodevice.

This special issue has received 84 papers from more than 10 countries and regions. Eight papers have been accepted to be published in this journal after reviewing by related reviewers and editor decision, which cover biomaterials and biological microdevices, micro- and nanofluidics, fabrication, metrology, solar cell technologies, nanomaterials, and nanoelectronics.

N. D. Israelsen et al., authors of the paper titled “Nanoparticle Properties and Synthesis Effects on Surface-Enhanced Raman Scattering Enhancement Factor: An Introduction,” provide an introduction to how the factors such as nanoparticle size, shape, material, configuration, Raman reporters, and protective coatings influence signal enhancement and how to optimize them during synthesis of SERS nanoparticles.

P. Koedrith et al., authors of the paper titled “Recent Trends in Rapid Environmental Monitoring of Pathogens and Toxicants: Potential of Nanoparticle-Based Biosensor and Applications,” describe current state of biosensor nanotechnology with regard to the developments over 27 conventional detection methods and the challenges due to reality of environmental 28 samples. Future directions to further develop novel detection devices and their advantages over other environmental monitoring methodologies are also discussed.

M. Yanagihara et al., authors of the paper titled “Vacuum Ultraviolet Field Emission Lamp Consisting of Neodymium Ion Doped Lutetium Fluoride Thin Film as Phosphor,” developed a vacuum ultraviolet (VUV) field emission lamp by using a neodymium ion doped lutetium fluoride (Nd^3+^:LuF_3_) thin film as solid-state phosphor and carbon nanofiber field electron emitters. The thin film was synthesized by pulsed laser deposition and incorporated into the lamp. The cathodoluminescence spectra of the lamp showed multiple emission peaks at 180, 225, and 255 nm. These emission spectra were in good agreement with the spectra reported for the Nd^3+^:LuF_3_ crystal. Moreover, application of an acceleration voltage effectively increased the emission intensity. These results contribute to the performance enhancement of the lamp operating in the VUV region.

M. Tajdidzadeh et al., authors of the paper titled “Synthesis of Silver Nanoparticles Dispersed in Various Aqueous Media Using Laser Ablation,” investigated the particle size, morphology, and stability of Ag-NPs. A Q-switched Nd: YAG pulsed laser (*λ* = 532 nm, 360 mJ/pulse) was used for ablation of a pure Ag plate for 30 min to prepare Ag-NPs in the organic compound such as ethylene glycol (EG) and biopolymer such as chitosan. The media (EG, chitosan) permitted the making of NPs with average size of Ag-NPs in EG is about 22 nm and in chitosan is about 10 nm in spherical form. Particle size, morphology, and stability of NPs were compared with distilled water as a reference. The stability of the samples was studied by measuring UV-visible absorption spectra of samples after one month. The result indicated that the formation efficiency of NPs in chitosan was higher than other media and NPs in chitosan solution were more stable than other media during one-month storage. This method for synthesis of silver NPs could be presented as a green method due to it is environmentally friendly nature.

B. Saifullah et al., authors of the paper titled “Development of a Highly Biocompatible Antituberculosis Nanodelivery Formulation Based on Para-Aminosalicylic Acid—Zinc Layered Hydroxide Nanocomposites,” developed a nanodelivery formulation based on para-aminosalicylic acid (PAS) and zinc layered hydroxide using zinc nitrate salt as a precursor. The developed formulation has fourfold higher efficacy of PAS against mycobacterium tuberculosis with minimum inhibitory concentration (MIC) of 1.40 *μ*g/mL compared to the free drug PAS with MIC of 5.0 *μ*g/mL. The newly developed formulation was also found active against bacteria Gram positive, Gram negative, and* Candida*. The formulation is also found biocompatible with human normal lung cells MRC-5 and mouse fibroblast cells 3T3. The in vitro release of PAS from the formulation was found sustained in human body simulated phosphate buffer saline (PBS) solution at pH of 7.4 and 4.8. Most importantly the nanocomposite prepared using zinc nitrate salt is advantageous in terms of yield, is free from toxic zinc oxide contamination, and has higher biocompatibility compared to the one prepared using zinc oxide precursor.

N. Soni and S. Prakash, authors of the paper titled “Green Nanoparticles for Mosquito Control,” used the green method for synthesis of silver and gold nanoparticles. The results were obtained using UV-visible spectrophotometer and the images were recorded with a transmission electron microscope (TEM). The efficacy tests were then performed at different concentrations and varying numbers of hours by probit analysis. The synthesized AgNPs were in spherical shape and average sizes (11.77 nm AgNPs, 46.48 nm AuNPs). The larvae of* A. stephensi* were found highly susceptible to the synthesized AgNPs and AuNPs than the* C. quinquefasciatus*. These results suggest that the* C. zeylanicum* synthesized silver and gold nanoparticles have the potential to be used as an ideal ecofriendly approach for the control of mosquito.

C. Zhao et al., authors of the paper titled “Photocatalytic Removal of Microcystin-LR by Advanced WO_3_-Based Nanoparticles under Simulated Solar Light,” synthesized a series of advanced WO_3_-based photocatalysts including CuO/WO_3_, Pd/WO_3_, and Pt/WO_3_ for the photocatalytic removal of microcystin-LR (MC-LR) under simulated solar light. In this study, Pt/WO_3_ exhibited the best performance for the photocatalytic degradation of MC-LR. The MC-LR degradation can be described by pseudo-first-order kinetic model. Chloride ion (Cl^−^) with proper concentration could enhance the MC-LR degradation. The presence of metal cations (Cu^2+^ and Fe^3+^) improved the photocatalytic degradation of MC-LR. This study suggests that Pt/WO_3_ photocatalytic oxidation under solar light is a promising option for the purification of water containing MC-LR.

W. H. Lee et al., authors of the paper titled “Self-Consolidation Mechanism of Nanostructured Ti_5_Si_3_ Compact Induced by Electrical Discharge,” applied electrical discharge using a capacitance of 450 *μ*F at 7.0 and 8.0 kJ input energies to mechanical alloyed Ti_5_Si_3_ powder without applying any external pressure. A solid bulk of nanostructured Ti_5_Si_3_ with no compositional deviation was obtained in times as short as 159 *μ*sec by the discharge. During an electrical discharge, the heat generated is the required parameter possibly to melt the Ti_5_Si_3_ particles and the pinch force can pressurize the melted powder without allowing the formation of pores. The following rapid cooling preserved the nanostructure of consolidated Ti_5_Si_3_ compact. Three stepped processes during an electrical discharge for the formation of nanostructured Ti_5_Si_3_ compact are proposed: (a) a physical breakdown of the surface oxide of Ti_5_Si_3_ powder particles, (b) melting and condensation of Ti_5_Si_3_ powder by the heat and pinch pressure, respectively, and (c) rapid cooling for the preservation of nanostructure. Complete conversion yielding a single phase Ti_5_Si_3_ is primarily dominated by the solid-liquid mechanism.

